# Mortality and cancer risk in patients with chronic pancreatitis in japan: insights into the importance of surveillance for pancreatic cancer

**DOI:** 10.1007/s00535-025-02321-0

**Published:** 2025-11-18

**Authors:** Ryotaro Matsumoto, Kazuhiro Kikuta, Tetsuya Takikawa, Yousuke Nakai, Mamoru Takenaka, Kentaro Oki, Eizaburo Ohno, Ken Ito, Nao Fujimori, Akio Katanuma, Atsuhiro Masuda, Yasuki Hori, Tsukasa Ikeura, Rei Suzuki, Satoshi Yamamoto, Yoshio Sogame, Hiroki Kawashima, Tetsuhide Ito, Kosuke Okuwaki, Takao Itoi, Yukiko Takayama, Akira Nakamura, Shuji Terai, Kazuyuki Matsumoto, Masaki Kuwatani, Masashi Kishiwada, Minoru Shigekawa, Tomoaki Matsumori, Osamu Inatomi, Waku Hatta, Atsushi Irisawa, Michiaki Unno, Yoshifumi Takeyama, Atsushi Masamune, Japan Pancreatitis Study Group for Chronic Pancreatitis, Masanori Gonda, Kazunao Hayashi, Yoshiki Hirooka, Hironari Kato, Masatoshi Murakami, Shuntaro Mukai, Shinji Nakayama, Kazunori Nakaoka, Naoki Okano, Nao Otsuka, Shuhei Shintani, Tadayuki Takagi, Yu Takamatsu, Kensuke Takuma, Keijiro Ueda, Masayuki Ueno, Takeji Umemura, Masafumi Watanabe, Akira Yamamiya, Satoki Yamane, Kentaro Yamao

**Affiliations:** 1 Division of Gastroenterology, Tohoku University Graduate School of Medicine, Sendai, Japan; 2 Department of Gastroenterology, Graduate School of Medicine, The University of Tokyo, Tokyo, Japan; 3 Department of Internal Medicine, Institute of Gastroenterology, Tokyo Women’s Medical University, Tokyo, Japan; 4 Department of Gastroenterology and Hepatology, Kindai University Faculty of Medicine, Osaka-Sayama, Japan; 5 Department of Gastroenterology and Hepatology, Kurashiki Central Hospital, Kurashiki, Japan; 6 Department of Gastroenterology and Hepatology, Fujita Health University School of Medicine, Nagoya, Japan; 7 Division of Gastroenterology and Hepatology, Toho University Omori Medical Center, Tokyo, Japan; 8 Department of Medicine and Bioregulatory Science, Graduate School of Medical Sciences, Kyushu University, Fukuoka, Japan; 9 Center for Gastroenterology, Teine-Keijinkai Hospital, Sapporo, Japan; 10 Division of Gastroenterology, Department of Internal Medicine, Kobe University Graduate School of Medicine, Kobe, Japan; 11 Department of Gastroenterology and Metabolism, Nagoya City University Graduate School of Medical Sciences, Nagoya, Japan; 12 Department of Gastroenterology and Hepatology, Kansai Medical University, Hirakata, Japan; 13 Department of Gastroenterology, Fukushima Medical University School of Medicine, Fukushima, Japan; 14 Department of Gastroenterology, Fujita Health University Bantane Hospital, Nagoya, Japan; 15 Department of Molecular Gastroenterology and Hepatology, Graduate School of Medical Science, Kyoto Prefectural University of Medicine, Kyoto, Japan; 16 Department of Gastroenterology and Hepatology, Nagoya University Graduate School of Medicine, Nagoya, Japan; 17 Neuroendocrine Tumor Centre, Fukuoka Sanno Hospital, International University of Health and Welfare, Fukuoka, Japan; 18 Department of Gastroenterology, Kitasato University School of Medicine, Sagamihara, Japan; 19 Department of Gastroenterology and Hepatology, Tokyo Medical University, Tokyo, Japan; 20 Department of Medicine, Division of Gastroenterology and Hepatology, Shinshu University School of Medicine, Matsumoto, Japan; 21 Division of Gastroenterology and Hepatology, Graduate School of Medical and Dental Sciences, Niigata University, Niigata, Japan; 22 Department of Gastroenterology and Hepatology, Okayama University Hospital, Okayama, Japan; 23 Department of Gastroenterology and Hepatology, Hokkaido University Hospital, Sapporo, Japan; 24 Department of Hepatobiliary Pancreatic and Transplant Surgery, Mie University Graduate School of Medicine, Tsu, Japan; 25 Department of Gastroenterology and Hepatology, The University of Osaka Graduate School of Medicine, Osaka, Japan; 26 Department of Gastroenterology and Hepatology, Kyoto University Graduate School of Medicine, Kyoto, Japan; 27 Department of Medicine, Shiga University of Medical Science, Otsu, Japan; 28 Department of Gastroenterology, Dokkyo Medical University School of Medicine, Mibu, Japan; 29 Department of Surgery, Tohoku University Graduate School of Medicine, Sendai, Japan; 30 Department of Surgery, Kindai University Faculty of Medicine, Osaka, Japan

**Keywords:** Alcohol, Chronic pancreatitis, Pancreatic cancer, Pancreatitis, Smoking

## Abstract

**Background/Objective:**

Since the 2010s, Japan’s national health insurance system has covered key management for chronic pancreatitis (CP), including pancreatic enzyme replacement therapy. These therapies are expected to improve long-term prognosis; however, recent data are lacking. This study aimed to clarify the updated cancer risk and mortality among patients with CP in Japan.

**Methods:**

We conducted a multicenter, retrospective cohort study on 1,110 patients with CP treated at 28 institutions in 2011. Standardized incidence ratios (SIRs) and standardized mortality ratios (SMRs) were calculated for comorbidities. Factors associated with the development of malignancy and overall survival were analyzed.

**Results:**

Patients with CP had an elevated SIR of 1.62 (95% confidence interval [CI], 1.43–1.83) for malignancy, with the highest risk observed for pancreatic cancer (SIR = 6.44 [95% CI, 4.64–8.90]). During follow-up, 143 patients (12.9%) died, most frequently from malignancy (47.5%). The SMR was elevated in all patients with CP (SMR = 1.20 [95% CI, 1.01–1.42]) and in those with alcohol-related CP (SMR = 1.49 [95% CI, 1.23–1.81]) but not in those with alcohol-unrelated CP. Pancreatic cancer was identified as the strongest factor associated with overall survival (hazard ratio, 48.92 in multivariate analysis). Overall survival of the patients with pancreatic cancer was significantly longer in those who underwent regular examinations for CP at least every three months (*P* = 0.011).

**Conclusions:**

Patients with alcohol-related CP have higher mortality than the general population in Japan. Pancreatic cancer remains a crucial prognostic factor in patients with CP. Regular surveillance for pancreatic cancer is important to improve their prognosis.

**Supplementary Information:**

The online version contains supplementary material available at 10.1007/s00535-025-02321-0.

## Introduction

Chronic pancreatitis (CP) is a progressive fibro-inflammatory syndrome of the pancreas influenced by genetic and environmental factors [1–4]. It is characterized by intractable abdominal and back pain, and, in advanced cases, it often leads to pancreatic exocrine insufficiency and diabetes mellitus. CP commonly results in malnutrition and alterations in body composition [5]. The latest nationwide epidemiological survey in Japan estimated that 56,520 individuals had CP, with a prevalence rate of 44.5 per 100,000 persons [6]. The mean age of the patients was 61.9 years. Previous studies have demonstrated that patients with CP experience increased mortality rates and reduced life expectancy [7–11]. In 1989, Levy et al. [7] reported that mortality in CP patients was 35.8% higher than that in the French population. A 30-year follow-up study from Denmark showed that the standardized mortality ratio (SMR) was 4.27 in male and 4.51 in female patients with CP [8]. In 1989, Miyake et al. [9] reported a higher mortality in Japanese patients with alcohol-related CP (by 26.2%), but not in those with alcohol-unrelated CP. A multicenter study conducted in 2006 on CP patients who were registered for the nationwide survey in 1994 found an increased SMR for all CP patients (SMR = 1.56) and for male patients (SMR = 1.72), but not for female patients (SMR = 0.94) [10]. Malignancies were the leading cause of death, accounting for 43.1% of all deaths, with an SMR of 2.01. Among malignancies, pancreatic cancer had the highest SMR, followed by liver cirrhosis (SMR = 3.64) and biliary cancer (SMR = 2.79). However, recent data on the mortality of patients with CP in Japan remain limited.

Previous studies have demonstrated an increased risk of malignancies in patients with CP. In a nationwide matched cohort study from Denmark, 13.6% of CP patients developed cancer, compared with 7.9% of controls, resulting in an adjusted hazard ratio (HR) of 1.2 [12]. Pancreatic and liver cancers had the highest excess risks among CP patients, with HRs of 6.9 and 2.0, respectively. CP is a well-established risk factor for pancreatic cancer [13–16]. According to a meta-analysis of seven studies, the risk of pancreatic cancer in patients with CP was 13.3 times higher than in the general population [13]. A Japanese study of 506 patients with CP who were followed for at least two years after diagnosis reported a standardized incidence ratio (SIR) of 11.8 for pancreatic cancer [14].

Since the 2010s, Japan’s national health insurance system has covered several key treatments for CP, including pancreatic enzyme replacement therapy (introduced in 2011), pancreatic stent placement (2012), and extracorporeal shock wave lithotripsy for pancreatic stones (2013). These therapies have been incorporated into the revised clinical guidelines for the management of CP in Japan [17] and are expected to improve the long-term prognosis of patients with CP. However, recent data are limited. This study aimed to update and clarify the risk of cancer and mortality among patients with CP in Japan.

## Methods

This study was conducted as a research project by members of the Pancreatitis Research Committee of the Japan Pancreas Society (the Japan Pancreatitis Study Group for Chronic Pancreatitis). We conducted a follow-up outcome survey of patients with definite CP who were treated at 28 participating institutions across Japan in 2011. The diagnosis of CP was based on the revised Japanese clinical diagnostic criteria proposed by the Japan Pancreas Society in 2009 [18].

### Data collection

We collected the following information from medical records: (a) Clinical characteristics, including age, etiology, date and age at CP diagnosis, date of last follow-up or death, and cause of death, if applicable. (b) Presence or absence of comorbidities after CP diagnosis, including malignancies, cardiovascular diseases, cerebrovascular diseases, pancreatic exocrine insufficiency, and diabetes mellitus, along with the date and age at diagnosis. The etiologies and clinical stages of CP were determined at the discretion of the treating physicians. Because of possible recall bias, underreporting, and fluctuations in actual alcohol and tobacco consumption [19–21], quantitative data on alcohol and tobacco use were not collected. Instead, information on drinking and smoking status was obtained. Pancreatic exocrine insufficiency was diagnosed based on clinical steatorrhea, the requirement for long-term oral pancreatic enzyme supplements, and/or abnormal results of the *N*-benzoyl-l-tyrosyl-*p*-aminobenzoic acid test. DM was diagnosed when any of the following criteria were met: fasting plasma glucose > 126 mg/dL, casual plasma glucose > 200 mg/dL, HbA1c ≥ 6.5%, or 2-h plasma glucose > 200 mg/dL during a 75-g oral glucose tolerance test [22]. Overall survival (OS) was defined as the time from the date of CP diagnosis to the date of the last follow-up or death. For patients who were diagnosed with pancreatic cancer after 2 years of CP diagnosis, we collected information on the principal clinical triggers for pancreatic cancer diagnosis, the intervals of blood tests and/or imaging studies performed on a regular basis, and the clinical stages of pancreatic cancer classified according to the 7th edition of the Japanese classification of pancreatic carcinoma by the Japan Pancreas Society [23].

### Statistical analysis

Statistical analyses were performed using JMP Pro 17.1.0 (SAS Institute, Cary, NC, USA) and R software version 4.1.2 (The R Foundation for Statistical Computing, Vienna, Austria). Categorical variables were presented as counts and percentages, while continuous variables were expressed as the mean and standard deviation (SD). The SMR and SIR were calculated by dividing the observed number of deaths or malignancies by the expected number. The expected number of cancer deaths or malignancies was estimated by multiplying the malignancy mortality or incidence rates in the standard population by the total person-years, stratified by calendar period, sex, and 5-year age group of the study cohort. The standard population was based on cancer mortality and incidence rates in Japan, as reported by the National Cancer Center, Japan (https://ganjoho.jp/reg_stat/statistics/data/dl/index.html#a14, accessed April 1, 2025). If a patient developed multiple malignancies, each was counted separately. Statistical significance was defined as a lower limit of the 95% confidence interval (CI) > 1.0 or an upper limit < 1.0. OS and cumulative incidence were estimated using Kaplan–Meier survival analysis and compared using the log-rank test. Univariate and multivariate stepwise Cox regression analyses were performed. Malignancies, including both pancreatic and non-pancreatic cancers, were included as time-dependent covariates. Candidate variables with a *P*-value < 0.10 in the univariate analysis were entered into a forward stepwise selection process, and selected variables were analyzed in the multivariate model. A two-sided *P*-value < 0.05 was considered statistically significant.

### Ethics

This study was conducted in accordance with the principles of the Declaration of Helsinki and was approved by the Ethics Committee of the Tohoku University Graduate School of Medicine (Approval No. 2023–1-1007). Due to the retrospective nature of the study, the Ethics Committee waived the requirement for informed consent. Instead, patients were informed about the study through the institution’s website and given the opportunity to opt out.

## Results

### Clinical characteristics of enrolled patients with CP

A total of 1,110 patients with CP were enrolled in this study (Table 1). The mean age in 2011 was 60.1 years, and the mean age at CP diagnosis was 55.8 years. Of these, 885 (79.7%) were male. The most common etiology was alcohol-related, followed by idiopathic cases. Regarding treatment, 326 patients (29.4%) received pancreatic enzyme replacement therapy, 500 (45.0%) underwent endoscopic therapy for CP, 264 (23.8%) underwent extracorporeal shock wave lithotripsy for pancreatic stones, and 124 (11.2%) underwent surgery for CP.

**Table 1:** Characteristics of the enrolled 1,110 patients with CP

Characteristics	n = 1,110
Age on July 1st, 2011	60.1 (13.6)
Age at diagnosis of CP, years, mean (SD)^a^	55.8 (14.2)
Period from diagnosis of CP, years, mean (SD)^b^	11.2 (7.0)
Age at diagnosis of CP ≥ 65 years, n (%)	305 (28.3)
Sex, male, n (%)	885 (79.7)
Etiology, n (%)^c^	
Alcohol-related	748 (76.4)
Idiopathic	168 (17.2)
Hereditary/Familial	22 (2.2)
Pancreas divisum	13 (1.3)
Autoimmune pancreatitis	10 (1.0)
Surgery	6 (0.6)
Intraductal papillary mucinous neoplasm	4 (0.4)
Pancreatic trauma	3 (0.3)
Others	5 (0.5)
Alcohol drinking status, n (%)^d^	
Current	365 (36.2)
Ever	360 (35.7)
Occasional	99 (9.8)
Never	184 (18.3)
Smoking status, n (%)^e^	
Current	332 (35.5)
Ever	374 (40.0)
Never	229 (24.5)
Clinical stages of CP, n (%)	
Compensated	402 (36.2)
Transitional	241 (21.7)
Decompensated	467 (42.1)
Pancreatic exocrine insufficiency, yes, n (%)	250 (22.5)
Diabetes mellitus, yes, n (%)	482 (43.4)
Endoscopic treatment for CP, n (%)	500 (45.0)
Extracorporeal shock wave lithotripsy for pancreatic stone, n (%)	264 (23.8)
Surgery for CP, n (%)	124 (11.2)
Cardiovascular diseases, yes, n (%)	117 (10.5)
Cerebrovascular diseases, yes, n (%)	85 (7.7)
Malignancies after the diagnosis of CP, yes, n (%)^#^	227 (20.5)
Pancreatic cancer after the diagnosis of CP, yes, n (%)	39 (3.5)
Number of deaths during follow-up, n (%)	143 (12.9)

Data were from ^a^1076, ^b^1072, ^c^979, and ^d^1008, and ^e^935cases

^#^: Fifteen patients were diagnosed with two malignancies, four with three, and one with four

CP, chronic pancreatitis; SD, standard deviation

Overall, 32 patients (2.9%) had a history of malignancy, with a total of 41 cases (Supplementary Table 1). Twenty-four patients had a single malignancy, seven had two, and one had three. Colorectal cancer was the most frequent (n = 11), followed by gastric cancer (n = 8) and prostate cancer (n = 6).

### Patients with CP had increased SIR for malignancies, particularly pancreatic cancer

A total of 253 malignancies were observed in 227 patients following a diagnosis of CP. Lung cancer (n = 41) was the most frequently observed malignancy, followed by pancreatic cancer (n = 39), gastric cancer (n = 27), and colorectal cancer (n = 22) (Table 2). Patients with CP had an increased risk of malignancies, with an SIR of 1.62 (95% CI, 1.43–1.83). The highest SIR was observed for pancreatic cancer (SIR = 6.44 [95% CI, 4.64–8.90]), with elevated risks also noted for oral and pharyngeal cancer, esophageal cancer, bladder cancer, bile duct cancer, liver cancer, and lung cancer compared to the age- and sex-matched general population. The SIR for alcohol- and smoking-related malignancies—such as oral and pharyngeal cancer, esophageal cancer, and lung cancer—was higher in alcohol-related CP but not in alcohol-unrelated CP (Supplementary Table 2). In contrast, the SIR for pancreatic cancer was elevated in both alcohol-related (SIR = 5.80 [95% CI, 3.73–8.94]) and alcohol-unrelated CP (SIR = 7.51 [95% CI, 4.52–12.30]), with a comparable risk in both groups.

**Table 2:** SIR for malignancies in patients with CP

Types of malignancies	Observed number	Expected number	SIR (95% CI)
All malignancies	253	156.46	1.62 (1.43–1.83)
Pancreatic cancer	39	6.06	6.44 (4.64–8.90)
Oral and pharyngeal cancer	18	3.84	4.69 (2.87–7.57)
Esophageal cancer	18	5.81	3.10 (1.89–5.01)
Bladder cancer	12	4.38	2.74 (1.48–4.93)
Bile duct cancer	9	3.58	2.51 (1.22–4.95)
Laryngeal cancer	3	1.34	2.25 (0.58–7.17)
Ovarian cancer	1	0.51	1.95 (0.10–12.67)
Liver cancer	16	8.67	1.85 (1.09–3.08)
Lung cancer	41	22.71	1.81 (1.32–2.48)
Malignant lymphoma	8	4.66	1.72 (0.80–3.54)
Breast cancer	6	3.91	1.53 (0.62–3.51)
Renal and urinary tract cancer	7	4.86	1.44 (0.63–3.11)
Leukemia	2	1.90	1.05 (0.18–4.24)
Gastric cancer	27	26.53	1.02 (0.69–1.51)
Colorectal cancer	22	24.30	0.91 (0.58–1.40)
Prostate cancer	15	21.43	0.70 (0.41–1.18)
Others	9	N/A	N/A

CI, confidence interval; CP, chronic pancreatitis; N/A, not available; SIR, standardized incidence ratio

The clinical stages of pancreatic cancer were distributed as follows: stage 0 (n = 2), IA (n = 5), IB (n = 2), IIA (n = 10), IIB (n = 3), III (n = 5), and IV (n = 12). When stratified by the timing of diagnosis, pancreatic cancer was identified in 7 patients within 1 year of CP diagnosis, 4 patients between 1 and 2 years, 6 patients between 2 and 5 years, 6 patients between 5 and 10 years, and 16 patients after 10 years. The SIR for pancreatic cancer was 29.73 within the first 2 years and 4.93 thereafter (3.80 between 2 and 5 years, and 5.37 after 5 years of CP diagnosis) (Supplementary Table 3).

We examined the follow-up strategy for CP of the 28 patients in whom pancreatic cancer was diagnosed after 2 years of CP diagnosis. Twenty-five patients (89.3%) underwent regular follow-up at the respective institutions, whereas three (10.7%) were followed at other institutions. The intervals of regular blood tests and/or imaging studies were every month (n = 5: 17.9%), every three months (n = 14: 50.0%), every six months (n = 6: 21.4%), every year (n = 2: 7.1%), and longer (n = 1, 3.6%). At the time of pancreatic cancer diagnosis, 21 patients (75.0%) were asymptomatic. The primary clinical triggers for pancreatic cancer diagnosis were symptoms in 7 patients (25.0%), abnormal laboratory findings (all involving elevated tumor markers) in 7 patients (25.0%), and abnormal imaging findings in 14 patients (50.0%) (Table 3). We compared the background characteristics of patients who underwent regular examinations every ≤ 3 months (n = 19) with those who were examined at longer intervals (n = 9). Patients with shorter examination intervals were younger at the time of CP diagnosis and had a higher proportion of endoscopic treatment compared with those with longer intervals (Supplementary Table 4).

**Table 3:** Primary clinical triggers for pancreatic cancer diagnosis

Primary clinical triggers	n (%)
Symptoms	7 (25.0)
Body weight loss	2 (7.1)
Jaundice	2 (7.1)
Pain	1 (3.6)
Fever	1 (3.6)
Anemia	1 (3.6)
Abnormal laboratory findings	7 (25.0)
Elevated CA19-9 level	4 (14.3)
Elevated CEA level	2 (7.1)^a^
Elevated DUPAN-2 level	1 (3.6)
Elevated liver enzyme levels	1 (3.6)^a^
Abnormal imaging findings	14 (50.0)
Appearance of pancreatic tumor	10 (35.7)^b^
Exacerbated MPD dilatation	6 (21.4)^b^
Increased size of pancreatic cyst	1 (3.6)

^a^One patient had elevated CEA and liver enzyme levels

^b^Three patients had both of these abnormal imaging findings

CA19-9, carbohydrate antigen; CEA, carcinoembryonic antigen; DUPAN-2,

Duke pancreatic monoclonal antigen type 2; MPD, main pancreatic duct

### Factors associated with malignancies and pancreatic cancer

We analyzed factors associated with the development of malignancies. In the multivariate analysis, age at CP diagnosis ≥ 65 years (HR = 2.38 [95% CI, 1.76–3.21]) and male sex (HR = 2.41 [95% CI, 1.54–3.79]) were identified as significant risk factors for malignancy (Table 4). Regarding pancreatic cancer, age at CP diagnosis ≥ 65 years was identified as a risk factor in the univariate analysis (Supplementary Table 5). In the univariate analysis, intervention for CP and endoscopic treatment showed a trend toward a reduced risk (*P* = 0.11 and *P* = 0.07, respectively), but the associations were not statistically significant. When cases of pancreatic cancer diagnosed within 2 years of CP diagnosis were excluded, age at CP diagnosis ≥ 65 years remained the only significant risk factor in the univariate analysis (Supplementary Table 6). Again, intervention for CP and endoscopic treatment showed a trend toward a reduced risk of pancreatic cancer, but the associations were not statistically significant (*P* = 0.12 and *P* = 0.13, respectively).

**Table 4:** Univariate and multivariate analysis of risk factors associated with malignancies in patients with CP

	Univariate analysis		Multivariate analysis	
	HR (95% CI)	*P* value	HR (95% CI)	*P* value
Age at diagnosis of CP ≥ 65 years	2.15 (1.60–2.89)	< 0.001	2.38 (1.76–3.21)	< 0.001
Sex, male	2.19 (1.42–3.38)	< 0.001	2.41 (1.54–3.79)	< 0.001
Etiology, alcohol-related	1.27 (0.94–1.72)	0.11		
Current alcohol drinking	1.29 (0.97–1.71)	0.09	1.26 (0.94–1.70)	0.12
Current smoking	1.07 (0.80–1.43)	0.67		
CP clinical stages^a^	1.01 (0.75–1.35)	0.95		
Pancreatic exocrine insufficiency	0.85 (0.61–1.18)	0.33		
Diabetes mellitus	1.10 (0.83–1.44)	0.51		

^a^Decompensated vs. other phases

CI, confidence interval; CP, chronic pancreatitis; HR, Hazard ratio

### Mortality

During the follow-up period, 143 patients (121 males and 22 females) died. The mean age at death was 68.9 years: 66.1 years for those with alcohol-related CP and 77.0 years for those with alcohol-unrelated CP (Supplementary Table 7). In comparison, the average life expectancy at birth in Japan in 2016 was 80.98 years for men and 87.14 years for women ((https://www.mhlw.go.jp/english/database/db-hw/lifetb16/dl/lifetb16-06.pdf)). Thus, the mean age at death in patients with CP appeared to be lower than that of the general Japanese population. The age at death was lower in patients with alcohol-related CP than in those with alcohol-unrelated CP, even when stratified by sex. The cause of death was identified in 122 patients. Malignancies were the leading cause, accounting for nearly half of the cases, followed by pneumonia (Supplementary Table 8).

We calculated the SMR for patients with CP. The overall SMR was 1.20 (95% CI, 1.01–1.42) compared to the age- and sex-matched general population (Table 5). The SMR was higher in alcohol-related CP (SMR = 1.49 [95% CI, 1.23–1.81]) but not in alcohol-unrelated CP (Supplementary Table 9). Among specific conditions, the highest SMR was observed for liver cirrhosis/failure (SMR = 5.03 [95% CI, 2.56–9.59]). When stratified by etiology, SMR was higher for malignancies, pneumonia, and liver cirrhosis/failure in alcohol-related CP, whereas no specific cause of death showed an elevated SMR in alcohol-unrelated CP. Pancreatic cancer was the only malignancy with a significantly elevated SMR in all patients with CP (SMR = 5.42 [95% CI, 3.48–8.36]) (Supplementary Table 10). Further stratification by etiology of CP revealed that the SMR was higher for pancreatic cancer, bile duct cancer, and oral and pharyngeal cancer in alcohol-related CP, whereas in alcohol-unrelated CP, only pancreatic cancer showed an elevated SMR (Supplementary Table 11).

**Table 5:** SMR in patients with CP

Causes of deaths	Observed number	Expected number	SMR (95% CI)
Total	143	119.00	1.20 (1.01–1.42)
Malignancies	58	47.53	1.22 (0.93–1.59)
Pneumonia	15	8.81	1.70 (0.99–2.88)
Liver cirrhosis/Liver failure	10	1.99	5.03 (2.56–9.59)
Infections	8	N/A	N/A
Cerebrovascular diseases	7	9.77	0.72 (0.32–1.56)
Cardiovascular diseases	6	16.52	0.42 (0.15–1.04)
Myelodysplastic syndromes	3	N/A	N/A
Trauma	3	3.80	0.79 (0.20–2.52)
Gastrointestinal bleeding	2	N/A	N/A
Intra-abdominal bleeding	2	N/A	N/A
Ileus	2	N/A	N/A
Renal failure	2	2.08	0.96 (0.17–3.87)
Acute pancreatitis	1	N/A	N/A
Hypoglycemia	1	N/A	N/A
Suicide	1	2.02	0.50 (0.03–3.25)
Senility	1	2.90	0.35 (0.02–2.27)
Unknown	21	N/A	N/A

CI, confidence interval; CP, chronic pancreatitis; N/A, not available; SMR, standardized mortality ratio

### Factors associated with OS

**Fig. 1:**
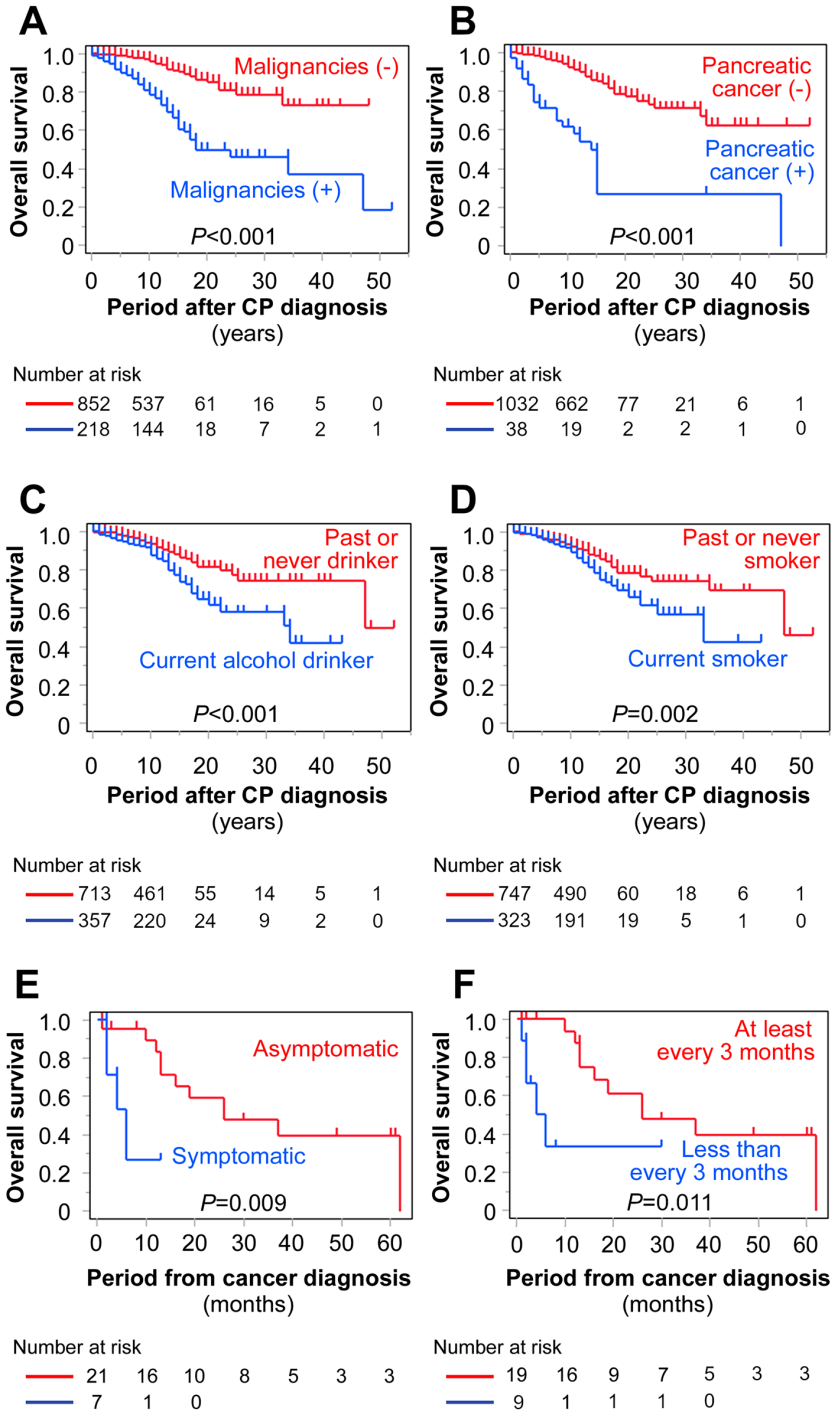
OS of patients with CP, estimated using Kaplan–Meier survival analysis. OS was compared according to the presence ( +) or absence (-) of malignancies (**A**) or pancreatic cancer (**B**), as well as alcohol consumption (**C**) and smoking status (**D**). Among patients with pancreatic cancer, OS was further compared based on the presence or absence of symptoms at diagnosis (**E**) and the frequency of regular examinations (at least every three months vs. less frequent) (**F**). Censored participants are indicated by tick marks

Finally, we analyzed the factors associated with OS in patients with CP. In a multivariate analysis, age at CP diagnosis ≥ 65 years, current alcohol consumption, cerebrovascular disease, and malignancies, including pancreatic cancer, were identified as risk factors for poor OS (Table 6). The HR for pancreatic cancer was as high as 48.92, indicating that pancreatic cancer is a critical prognostic factor in patients with CP. We compared the survival curves of patients diagnosed with malignancies and pancreatic cancer to those without these diseases. The OS rate was significantly lower in patients with malignancies than in those without (*P* < 0.001) (Fig. 1). Similarly, the OS rate was significantly lower in patients with pancreatic cancer than in those without (*P* < 0.001). The OS rates were lower in patients who currently consume alcohol or smoke compared to those who do not, with *P*-values of < 0.001 for alcohol consumption and 0.002 for smoking status. The OS rate of patients with pancreatic cancer was higher in those diagnosed asymptomatically compared to those with symptoms (*P* = 0.009). Regarding the frequency of regular examinations for CP, the OS rate was higher in patients who underwent regular examinations (at least a blood test) every three months or more frequently, compared to those who underwent them less frequently (*P* = 0.011).

**Table 6:** Uni- and multivariate analysis of risk factors associated with OS in patients with CP

	Univariate analysis		Multivariate analysis	
	HR (95% CI)	*P* value	HR (95% CI)	*P* value
Age at diagnosis of CP ≥ 65 years	2.27 (1.58–3.26)	< 0.001	2.34 (1.58–3.46)	< 0.001
Gender, male	1.67 (1.04–2.70)	0.034	1.08 (0.64–1.82)	0.77
Etiology, alcohol-related	1.61 (1.09–2.38)	0.016	1.05 (0.64–1.71)	0.85
Current alcohol drinking	2.03 (1.45–2.84)	< 0.001	2.03 (1.34–3.08)	< 0.001
Current smoking	1.69 (1.20–2.38)	0.003	1.44 (0.95–2.18)	0.088
CP clinical stages^a^	1.03 (0.74–1.43)	0.88		
Pancreatic exocrine insufficiency	0.97 (0.66–1.43)	0.88		
Diabetes mellitus	1.15 (0.82–1.60)	0.43		
Cardiovascular diseases	1.53 (0.97–2.39)	0.06		
Cerebrovascular diseases	2.02 (1.26–3.26)	0.004	2.20 (1.29–3.74)	0.004
Malignancies	9.97 (6.73–14.78)	< 0.001		
Pancreatic cancer	31.40 (15.58–63.29)	< 0.001	48.92 (25.25–94.75)	< 0.001
Non-pancreatic malignancies	6.39 (4.26–9.60)	< 0.001	6.02 (3.84–9.44)	< 0.001

^a^Decompensated vs. other phases

CI, confidence interval; CP, chronic pancreatitis; HR, hazard ratio; OS, overall survival

## Discussion

The major findings of this study are as follows. First, patients with CP have a higher SIR for malignancies, with pancreatic cancer being the highest. SIR for pancreatic cancer was particularly high at 29.73 within two years after the diagnosis of CP. Second, age at diagnosis of CP ≥ 65 years and male sex were identified as independent risk factors for pancreatic cancer in a multivariate analysis. Third, SMR was higher in alcohol-related but not in alcohol-unrelated CP. Compared with the previous nationwide multicenter survey conducted in Japan in 2006, the SMR appeared to have decreased [10]. Fourth, among patients with pancreatic cancer, OS was better in those who underwent regular examinations every three months or more frequently compared to those who did not. Lastly, pancreatic cancer was the leading cause of death among malignancies and was identified as the most significant factor associated with overall survival in patients with CP. This study provides updated evidence on the risk of cancer and mortality among patients with CP in Japan.

Previous studies have shown that patients with CP have an increased risk of malignancies. Bang et al. [12], using data from the Danish National Patient Registry, reported a higher risk of malignancies (HR = 1.2), including cancers of the esophagus, small intestine, liver, pancreas, and lung, in patients with CP compared with controls. Han et al. [24] showed that patients with CP had a higher risk of all malignancies (HR = 1.2), esophageal cancer (HR = 3.9), and pancreatic cancer (HR = 3.9) using national claims data in South Korea. Pedrazzoli et al. [25] found a higher incidence of smoking-related tumors, such as lung, esophageal, oral, and pharyngeal cancers, in patients with CP. These results suggest that patients with CP are at an increased risk for malignancies, especially those related to alcohol consumption and smoking. In line with these findings, our study demonstrated that SIR for malignancies was 1.77 in alcohol-related CP but 1.34 in alcohol-unrelated CP. SIRs for alcohol- and smoking-related malignancies such as oral and pharyngeal, esophageal, and lung cancers were higher in alcohol-related CP but not in alcohol-unrelated CP. These findings underscore the importance of cancer surveillance targeting these organs in patients with alcohol-related CP.

Although patients with CP have higher mortality than the general population, the magnitude of this increase, ranging from approximately fivefold [12] to 1.25-fold [20] compared with the general population. These variations can be attributed to several factors, including differences in study periods, populations, etiologies, interventions, study designs, and adjustment variables (including the Charlson comorbidity index and socioeconomic status). In general, patients with alcohol-related CP have higher mortality than those with alcohol-unrelated CP. Miyake et al. [9] reported higher mortality in Japanese patients with alcohol-related CP (by 26.2%) but not in those with alcohol-unrelated CP. The SMR was increased to 2.7 in patients with moderate to severe alcohol consumption, whereas it was not increased in those with none or minimal consumption [26]. These findings are consistent with the more severe natural course observed in alcohol-related CP, which includes the development of diabetes, steatorrhea, pancreatic pseudocysts, pancreatic stones, and biliary stricture, compared to idiopathic cases [27]. Additionally, as mentioned earlier, patients with alcohol-related CP have higher SIR for malignancies compared to those with alcohol-unrelated CP. Continued alcohol consumption and smoking are known to be poor prognostic factors influencing mortality in patients with CP [13, 25, 27]. A Japanese study demonstrated that abstinence from alcohol reduced the risk of pancreatic cancer by five-fold [14]. Since the majority of mortality in CP patients is attributed to extrapancreatic consequences of alcohol and smoking overuse [28], lifestyle modifications, particularly alcohol abstinence and smoking cessation, are essential for improving survival outcomes in patients with alcohol-related CP.

In the previous multicenter study conducted in 2006 on patients with CP registered in 1994, the SMR was elevated in all patients and in males, but not in females [10]. Notably, the SMR decreased from 1.56 to 1.20 in all patients with CP, and from 1.72 to a statistically nonsignificant 1.20 in males. This reduction in SMR, particularly from 2.01 to 1.22 for malignancies, may have contributed substantially to the overall decrease in SMR. However, since the previous study did not report the SIR for malignancies [10], it remains unclear whether the decreased SMR is due to a reduced incidence of malignancies or due to earlier diagnosis. Furthermore, although there is no direct evidence, these improvements might be attributed to recent advances in the management of CP, such as pancreatic enzyme replacement therapy, which is now covered by Japan’s national health insurance. Pancreatic exocrine insufficiency has been identified as an independent risk factor for cardiovascular events and mortality [29], and the absence of pancreatic enzyme replacement therapy has been shown to be an independent predictor of increased postoperative mortality in patients undergoing surgery for CP [30].

CP is a well-established risk factor for pancreatic cancer. A recent systematic review and meta-analysis reported that the risk estimates for pancreatic cancer at 2, 5, and 9 years after CP diagnosis were 16.16, 7.90, and 3.53, respectively [15]. In this study, the SIR for pancreatic cancer was 6.44 across all observation periods and 4.93 after 2 years. The highest risk of pancreatic cancer was observed within the first 2 years (SIR = 29.73), suggesting that some pancreatic cancers are initially missed at the time of CP diagnosis. Munigala et al. [31] suggested that approximately 5% of pancreatic cancer cases are initially misdiagnosed as CP, with a potential diagnostic delay of up to 2 years. Therefore, it is crucial to remain vigilant and not overlook the possibility of pancreatic cancer at or shortly after the CP diagnosis, particularly in cases with ductal dilatation but without calcification. Such cases should be thoroughly investigated to exclude small periampullary tumors that may not be visible on cross-sectional imaging [32].

In a recent nationwide population-based cohort study from Denmark, the cumulative incidence of pancreatic cancer after a 2-year latency period was 1% during a mean follow-up period of 16.8 years [33], indicating that the long-term absolute risk is low. In our study, more than half of the pancreatic cancer cases were diagnosed at resectable stages (up to stage IIB) after the diagnosis of CP. The OS of patients with asymptomatic pancreatic cancer was longer than that of those with symptomatic disease, consistent with our previous findings [34]. Notably, among patients diagnosed with pancreatic cancer, those who underwent regular CP follow-up examinations every 3 months or more frequently had better OS than those who did not, underscoring the importance of regular surveillance for early pancreatic cancer detection. To date, a standardized screening strategy for pancreatic cancer in patients with sporadic CP has not been established [35], and no studies have conclusively demonstrated that surveillance improves the prognosis in these patients [36]. Indeed, the standard follow-up intervals varied among the 28 participating institutions: blood tests were performed monthly at one institution, every 2–3 months at two institutions, every 3 months at 16 institutions, every 4 months at one institution, and every 6 months at seven institutions. Imaging studies (most commonly computed tomography and magnetic resonance imaging/magnetic resonance cholangiopancreatography, performed alternately) were conducted every 6 months at 18 institutions and every 12 months at 10 institutions. Identifying an optimal subgroup for surveillance is therefore crucial. Kim et al. [37] identified age over 60 years and serum CA 19–9 levels greater than 100 U/mL as independent risk factors for pancreatic cancer in CP patients. Implementing structured surveillance programs incorporating biomarkers such as CA 19–9 and imaging modalities could potentially reduce mortality and improve outcomes in these patients.

Previous studies have suggested that early surgical intervention for CP may help prevent the development of pancreatic cancer [14, 38]. A multicenter retrospective study in Japan involving 506 patients with CP found that the SIR for pancreatic cancer was 11.8 after excluding cases diagnosed within the first 2 years following CP diagnosis. Over a median follow-up period of more than 5 years, the incidence of pancreatic cancer was lower in patients who underwent surgery (1/147, 0.7%) than in those who did not (18/352, 5.1%). The authors concluded that surgery for CP reduced the risk of pancreatic cancer by approximately tenfold. Similarly, Zheng et al. [38] analyzed 650 patients with surgically managed CP and found that the interval between CP diagnosis and surgery was shorter in patients who did not develop pancreatic cancer, suggesting that early surgical intervention may prevent pancreatic carcinogenesis in patients with CP. The proposed mechanisms underlying this protective effect include the relief of compartment syndrome and inflammation through surgical drainage or resection of fibrotic pancreatic parenchyma, as persistent, uncontrolled inflammation is a well-recognized risk factor for pancreatic carcinogenesis [38, 39]. Given these findings, it is worth investigating whether endoscopic treatments—such as pancreatic duct stenting or stone removal—could similarly reduce the risk of pancreatic cancer by alleviating pancreatic compartment syndrome. In the present study, although endoscopic treatment tended to be associated with a reduced risk of pancreatic cancer in CP patients (*P* = 0.07), it was not statistically significant.

This study has several limitations. First, it was a retrospective observational study conducted on patients managed in 2011, which may introduce recall bias, and some data were missing. Second, although the SMR was calculated using sex- and age-matched controls, adjustments for comorbidities and socioeconomic status were not possible because relevant data were unavailable. Third, detailed information on laboratory and imaging findings, pancreatic enzyme replacement therapy, and interventions was lacking. Fourth, although our findings suggest that regular examinations may facilitate the early diagnosis of pancreatic cancer, examination protocols were not standardized, and the type and frequency of examinations were left to the discretion of the treating physicians. Lastly, all patients in this study were managed by specialists in pancreatitis care, which may not reflect routine practice in general hospitals across Japan. Consequently, mortality rates might have been underestimated owing to the comprehensive care provided. Despite these limitations, our study provides updated data on cancer risk and mortality among patients with CP in Japan, based on a large cohort of more than 1,000 individuals.

## Conclusions

Patients with CP have higher mortality and an increased risk of malignancies, particularly pancreatic cancer, compared with the general population. Those with alcohol-related CP are at greater risk of extra-pancreatic malignancies, underscoring the importance of lifestyle modifications such as abstinence from alcohol and smoking cessation. Regular follow-up may be essential for the surveillance of pancreatic cancer. Further studies are warranted to establish effective screening and surveillance strategies for the early detection of pancreatic cancer and to improve the prognosis of patients with CP.

## Supplementary Information

Below is the link to the electronic supplementary material.

Supplementary file1 (DOCX 54 KB)

## Abbreviations

CA19-9: Carbohydrate antigen 19–9
CI: Confidence interval
CP: Chronic pancreatitis
HR: Hazard ratio
OS: Overall survival
SD: Standard deviation
SIR: Standardized incidence ratio
SMR: Standardized mortality ratio
