# Supplementary material for: Mortality and cancer risk in patients with chronic pancreatitis in japan: insights into the importance of surveillance for pancreatic cancer
Source: J Gastroenterol. 2025 Nov 18;61(1):105–16. doi: 10.1007/s00535-025-02321-0 (PMC12791062; doi:10.1007/s00535-025-02321-0)
Supplement: Supplementary file 1 — Supplementary file1 (DOCX 54 KB) [file 535_2025_2321_MOESM1_ESM.docx]

| **Supplementary Table 1. Past history of malignancies** | |
| --- | --- |
| Types of malignancies | n |
| All malignancies | 41 |
| Colorectal cancer | 11 |
| Gastric cancer | 8 |
| Prostate cancer | 6 |
| Lung cancer | 3 |
| Oral and pharyngeal cancer | 2 |
| Esophageal cancer | 2 |
| Liver cancer | 2 |
| Renal and urinary tract cancer | 2 |
| Biliary cancer | 1 |
| Bladder cancer | 1 |
| Ovarian cancer | 1 |
| Thyroid cancer | 1 |
| Malignant lymphoma | 1 |
| Twenty-four patients had one malignancy, seven had two, and one had three. | |

| **Supplementary Table 2. SIR for malignancies in patients with alcohol-related or alcohol-unrelated CP** | | | | | | | |
| --- | --- | --- | --- | --- | --- | --- | --- |
|  | Alcohol-related | | |  | Alcohol-unrelated | | |
| Types of malignancies | Observed number | Expected number | SIR (95% CI) |  | Observed number | Expected number | SIR (95% CI) |
| All malignancies | 180 | 101.92 | 1.77 (1.53–2.05) |  | 73 | 54.54 | 1.34 (1.06–1.69) |
| Pancreatic cancer | 22 | 3.80 | 5.80 (3.73–8.94) |  | 17 | 2.26 | 7.51 (4.52–12.30) |
| Oral and pharyngeal cancer | 15 | 2.68 | 5.60 (3.25–9.47) |  | 3 | 1.16 | 2.58 (0.67–8.22) |
| Esophageal cancer | 15 | 4.10 | 3.66 (2.13–6.19) |  | 3 | 1.70 | 1.76 (0.45–5.61) |
| Bladder cancer | 6 | 2.91 | 2.06 (0.84–4.73) |  | 6 | 1.47 | 4.09 (1.66–9.39) |
| Bile duct cancer | 5 | 2.14 | 2.33 (0.86–5.78) |  | 4 | 1.44 | 2.77 (0.89–7.62) |
| Laryngeal cancer | 3 | 0.95 | 3.15 (0.81–10.04) |  | 0 | 0.38 | N/A |
| Ovarian cancer | 1 | 0.17 | 5.78 (0.30–37.54) |  | 0 | 0.34 | N/A |
| Liver cancer | 10 | 5.70 | 1.75 (0.89–3.33) |  | 6 | 2.97 | 2.02 (0.82–4.64) |
| Lung cancer | 35 | 14.95 | 2.34 (1.65–3.29) |  | 6 | 7.76 | 0.77 (0.31–1.77) |
| Malignant lymphoma | 6 | 3.00 | 2.00 (0.81–4.59) |  | 2 | 1.66 | 1.21 (0.21–4.88) |
| Breast cancer | 2 | 1.35 | 1.48 (0.26–5.97) |  | 4 | 2.56 | 1.53 (0.49–4.21) |
| Renal and urinary tract cancer | 4 | 3.29 | 1.21 (0.39–3.33) |  | 3 | 1.56 | 1.92 (0.50–6.12) |
| Leukemia | 2 | 1.25 | 1.61 (0.28–6.49) |  | 0 | 0.65 | N/A |
| Gastric cancer | 22 | 17.66 | 1.25 (0.80–1.93) |  | 5 | 8.87 | 0.56 (0.21–1.39) |
| Colorectal cancer | 17 | 15.87 | 1.07 (0.64-1.75) |  | 5 | 8.43 | 0.59 (0.22-1.46) |
| Prostate cancer | 9 | 14.98 | 0.60 (0.29-1.18) |  | 6 | 6.46 | 0.93 (0.38-2.14) |
| Others | 6 | N/A | N/A |  | 3 | N/A | N/A |
| CI, confidence interval; CP, chronic pancreatitis; N/A, not available; SIR, standardized incidence ratio | | | | | | | |

| **Supplementary Table 3. SIR for pancreatic cancer according to the interval from CP diagnosis** | | | | |
| --- | --- | --- | --- | --- |
| Interval from CP diagnosis | Person-years | Observed number | Expected number | SIR (95% CI) |
| All period | 11510 | 39 | 6.06 | 6.44 (4.64–8.90) |
| <2 years | 1035 | 11 | 0.37 | 29.73 (15.64–54.97) |
| ≥2 years | 10475 | 28 | 5.68 | 4.93 (3.34–7.23) |
| ≥2 to <5 years | 3716 | 6 | 1.58 | 3.80 (1.54–8.72) |
| ≥5 years | 6759 | 22 | 4.10 | 5.37 (3.45–8.28) |
| CI, confidence interval; CP, chronic pancreatitis; SIR, standardized incidence ratio | | | | |

| **Supplementary Table 4. Comparison of characteristics between patients with pancreatic cancer under surveillance every ≤3 months and those under longer surveillance intervals** | | | |
| --- | --- | --- | --- |
|  | Interval of surveillance | |  |
|  | **≤**3 months (n=19) | >3 months (n=9) | *P* value |
| Age on July 1^st^, 2011, years, mean (SD) | 66.7 (9.1) | 70.8 (5.6) | 0.23 |
| Age at diagnosis of CP, years, mean (SD)^a^ | 59.8 (9.4) | 68.0 (7.0) | 0.028 |
| Age at diagnosis of pancreatic cancer, years, mean (SD) | 71.9 (8.8) | 76.9 (4.4) | 0.13 |
| Interval between diagnoses of CP and pancreatic cancer, years, mean (SD)^b^ | 12.2 (10.2) | 8.9 (4.4) | 0.37 |
| Sex, male, n (%) | 15 (78.9) | 6 (66.7) | 0.48 |
| Etiology, n (%)^c^ |  |  | 0.69 |
| Alcohol-related | 11 (57.9) | 4 (44.4) |  |
| Idiopathic | 8 (42.1) | 5 (55.6) |  |
| Alcohol drinking status, n (%)^d^ |  |  | 0.41 |
| Current | 5 (26.3) | 2 (22.2) |  |
| Ever | 6 (31.6) | 2 (22.2) |  |
| Occasional | 5 (26.3) | 1 (11.1) |  |
| Never | 3 (15.8) | 4 (44.4) |  |
| Smoking status, n (%)^e^ |  |  | 0.86 |
| Current | 6 (31.6) | 2 (22.2) |  |
| Ever | 8 (42.1) | 4 (44.4) |  |
| Never | 5 (26.3) | 3 (33.3) |  |
| CP clinical stages, n (%) |  |  | 0.59 |
| Compensated | 4 (21.1) | 4 (44.4) |  |
| Transitional | 3 (15.8) | 2 (22.2) |  |
| Decompensated | 12 (63.2) | 3 (33.3) |  |
| Pancreatic exocrine insufficiency, yes, n (%) | 8 (42.1) | 2 (22.2) | 0.42 |
| Diabetes mellitus, yes, n (%) | 12 (63.2) | 4 (44.4) | 0.35 |
| Endoscopic treatment for CP, n (%) | 8 (42.1) | 0 (0) | 0.021 |
| Extracorporeal shock wave lithotripsy for pancreatic stone, n (%) | 4 (21.1) | 0 (0) | 0.14 |
| Surgery for CP, n (%) | 3 (15.8) | 1 (11.1) | 0.74 |
| Primary clinical trigger for pancreatic cancer diagnosis, n (%) |  |  | 0.035 |
| Symptoms | 2 (10.5) | 5 (55.5) |  |
| Abnormal laboratory findings | 6 (31.6) | 1 (11.1) |  |
| Abnormal imaging findings | 11 (57.9) | 3 (33.3) |  |
| Clinical stages of pancreatic cancer, n (%) |  |  | 0.83 |
| 0 | 2 (10.5) | 0 (0) |  |
| IA | 3 (15.8) | 1 (11.1) |  |
| IB | 1 (5.3) | 1 (11.1) |  |
| IIA | 5 (26.3) | 1 (11.1) |  |
| IIB | 1 (5.3) | 1 (11.1) |  |
| III | 1 (5.3) | 1 (11.1) |  |
| IV | 6 (31.6) | 4 (44.4) |  |
| CP, chronic pancreatitis; SD, standard deviation | | | |

| **Supplementary Table 5. Uni- and multivariate analysis of risk factors associated with pancreatic cancer in patients with CP** | | | | | |
| --- | --- | --- | --- | --- | --- |
|  | Univariate analysis | |  | Multivariate analysis | |
|  | HR (95% CI) | *P* value |  | HR (95% CI) | *P* value |
| Age at diagnosis of CP ≥65 years | 2.04 (1.04–4.01) | 0.038 |  | 1.83 (0.92–3.66) | 0.09 |
| Sex, male | 1.33 (0.58–3.06) | 0.50 |  |  |  |
| Etiology, alcohol-related | 0.67 (0.35–1.26) | 0.21 |  |  |  |
| Current alcohol drinking | 0.75 (0.36–1.55) | 0.44 |  |  |  |
| Current smoking | 0.88 (0.43–1.81) | 0.72 |  |  |  |
| CP clinical stages^a^ | 1.20 (0.59–2.44) | 0.61 |  |  |  |
| Pancreatic exocrine insufficiency | 0.59 (0.25–1.42) | 0.24 |  |  |  |
| Diabetes mellitus | 1.43 (0.76–2.70) | 0.27 |  |  |  |
| Endoscopic treatment for CP | 0.57 (0.29–1.13) | 0.11 |  |  |  |
| Surgery for CP | 0.82 (0.31–2.18) | 0.69 |  |  |  |
| Intervention for CP | 0.55 (0.29–1.05) | 0.07 |  | 0.64 (0.33–1.26) | 0.20 |
| ^a^Decompensated vs. other phases  CI, confidence interval; CP, chronic pancreatitis; HR, Hazard ratio | | | | | |

| **Supplementary Table 6. Univariate analysis of risk factors associated with the diagnosis of pancreatic cancer in patients with CP (excluding cases diagnosed within 2 years after CP diagnosis)** | | |
| --- | --- | --- |
|  | Univariate analysis | |
|  | HR (95% CI) | *P* value |
| Age at diagnosis of CP ≥65 years | 2.59 (1.18–5.66) | 0.018 |
| Sex, male | 0.91 (0.38–2.18) | 0.83 |
| Etiology, alcohol-related | 0.62 (0.29–1.30) | 0.21 |
| Current alcohol drinking | 0.61 (0.25–1.52) | 0.29 |
| Current smoking | 1.06 (0.46–2.42) | 0.89 |
| Pancreatic exocrine insufficiency | 0.53 (0.18–1.54) | 0.24 |
| Diabetes mellitus | 1.38 (0.65–2.93) | 0.40 |
| Endoscopic treatment for CP | 0.52 (0.23–1.20) | 0.13 |
| Surgery for CP | 0.84 (0.28–2.55) | 0.76 |
| Intervention for CP | 0.55 (0.26–1.17) | 0.12 |
| CI, confidence interval; CP, chronic pancreatitis; HR, Hazard ratio | | |

| **Supplementary Table 7. Age at death in patients with CP** | | | | |
| --- | --- | --- | --- | --- |
|  | All CP  (n = 143) | Alcohol-related  (n = 106) | Alcohol-unrelated (n = 37) | *P* value |
| Males, mean (SD), years | 69.0 (11.6) | 67.1 (11.3) | 76.8 (9.2) | <0.001 |
| Females, mean (SD), years | 68.5 (16.3) | 55.4 (8.9) | 77.5 (14.1) | <0.001 |
| All CP, mean (SD) | 68.9 (12.4) | 66.1 (11.6) | 77.0 (11.0) | <0.001 |
| During the follow-up period, 121 male patients with CP (97 alcohol-related and 24 alcohol-unrelated) and 22 female patients (9 alcohol-related and 13 alcohol-unrelated) died.  CP, chronic pancreatitis; SD, standard deviation | | | | |

| **Supplementary Table 8. Causes of death in 122 patients with CP** | |  |
| --- | --- | --- |
| Causes of death | n (%) |  |
| Malignancies | 58 (47.5) |  |
| Pneumonia | 15 (12.3) |  |
| Liver cirrhosis/Liver failure | 10 (8.2) |  |
| Infection | 8 (6.6) |  |
| Cerebrovascular diseases | 7 (5.7) |  |
| Cardiovascular diseases | 6 (4.9) |  |
| Myelodysplastic syndromes | 3 (2.5) |  |
| Trauma | 3 (2.5) |  |
| Gastrointestinal bleeding | 2 (1.6) |  |
| Intra-abdominal bleeding | 2 (1.6) |  |
| Ileus | 2 (1.6) |  |
| Renal failure | 2 (1.6) |  |
| Acute pancreatitis | 1 (0.8) |  |
| Hypoglycemia | 1 (0.8) |  |
| Suicide | 1 (0.8) |  |
| Senility | 1 (0.8) |  |
| The cause of death was unknown in 21 patients. | |  |

| **Supplementary Table 9. SMR in patients with alcohol-related and alcohol-unrelated CP** | | | | | | | |
| --- | --- | --- | --- | --- | --- | --- | --- |
|  | Alcohol-related CP | | |  | Alcohol-unrelated CP | | |
|  | Observed number | Expected number | SMR (95% CI) |  | Observed number | Expected number | SMR (95% CI) |
| Total | 106 | 71.20 | 1.49 (1.23–1.81) |  | 37 | 47.81 | 0.77 (0.55–1.07) |
| Malignancies | 42 | 29.71 | 1.41 (1.03–1.93) |  | 16 | 17.82 | 0.90 (0.53–1.50) |
| Pneumonia | 12 | 5.03 | 2.39 (1.30–4.30) |  | 3 | 3.78 | 0.79 (0.20–2.52) |
| Liver cirrhosis/Liver failure | 9 | 1.35 | 6.68 (3.26–13.18) |  | 1 | 0.64 | 1.56 (0.08–10.13) |
| Infections | 8 | N/A | N/A |  | 0 | N/A | N/A |
| Cerebrovascular diseases | 2 | 5.67 | 0.35 (0.06–1.41) |  | 5 | 4.10 | 1.22 (0.45–3.03) |
| Cardiovascular diseases | 4 | 9.61 | 0.42 (0.13–1.16) |  | 2 | 6.92 | 0.29 (0.05–1.17) |
| Myelodysplastic syndromes | 3 | N/A | N/A |  | 0 | N/A | N/A |
| Trauma | 0 | 2.48 | N/A |  | 3 | 1.47 | 2.04 (0.53–6.50) |
| Gastrointestinal bleeding | 2 | N/A | N/A |  | 0 | N/A | N/A |
| Intra-abdominal bleeding | 2 | N/A | N/A |  | 0 | N/A | N/A |
| Ileus | 0 | N/A | N/A |  | 2 | N/A | N/A |
| Renal failure | 1 | 1.17 | 0.86 (0.04–5.59) |  | 1 | 0.91 | 1.10 (0.05–5.91) |
| Acute pancreatitis | 1 | N/A | N/A |  | 0 | N/A | N/A |
| Hypoglycemia | 1 | N/A | N/A |  | 0 | N/A | N/A |
| Suicide | 1 | 1.45 | 0.69 (0.04–4.48) |  | 0 | 0.57 | N/A |
| Senility | 1 | 1.25 | 0.80 (0.04–5.20) |  | 0 | 1.65 | N/A |
| Unknown | 17 | N/A | N/A |  | 4 | N/A | N/A |
| CI, confidence interval; CP, chronic pancreatitis; N/A, not available; SMR, standardized mortality ratio | | | | | | | |

| **Supplementary Table 10. SMR for malignancies in patients with CP** | | | |
| --- | --- | --- | --- |
| Types of malignancies | Observed number | Expected number | SMR (95% CI) |
| All malignancies | 58 | 47.53 | 1.22 (0.93–1.59) |
| Pancreatic cancer | 22 | 4.06 | 5.42 (3.48–8.36) |
| Lung cancer | 13 | 11.00 | 1.18 (0.66–2.08) |
| Bile duct cancer | 5 | 2.04 | 2.45 (0.90–6.08) |
| Liver cancer | 5 | 4.03 | 1.24 (0.46–3.08) |
| Oral and pharyngeal cancer | 3 | 1.15 | 2.61 (0.67–8.32) |
| Colorectal cancer | 3 | 6.13 | 0.49 (0.13–1.56) |
| Esophageal cancer | 2 | 2.04 | 0.98 (0.17–3.95) |
| Renal and urinary tract cancer | 2 | 1.27 | 1.57 (0.27–6.33) |
| Gastric cancer | 1 | 6.34 | 0.16 (0.01–1.04) |
| Others | 2 | N/A | N/A |
| CI, confidence interval; CP, chronic pancreatitis; N/A, not available; SMR, standardized mortality ratio | | | |

|  | **Supplementary Table 11. SMR for malignancies in patients with alcohol-related or alcohol-unrelated CP** | | | | | | | |
| --- | --- | --- | --- | --- | --- | --- | --- | --- |
|  | | Alcohol-related CP | | |  | Alcohol-unrelated CP | | |
|  | | Observed number | Expected number | SMR (95% CI) |  | Observed number | Expected number | SMR (95% CI) |
| Types of malignancies | | 42 | 29.71 | 1.41 (1.03-1.93) |  | 16 | 17.82 | 0.90 (0.53-1.50) |
| Pancreatic cancer | | 13 | 2.47 | 5.26 (2.93-9.26) |  | 9 | 1.59 | 5.66 (2.6-11.17) |
| Lung cancer | | 10 | 7.10 | 1.41 (0.72-2.69) |  | 3 | 3.90 | 0.77 (0.20-2.45) |
| Bile duct cancer | | 4 | 1.17 | 3.43 (1.10-9.43) |  | 1 | 0.88 | 1.14 (0.05-5.72) |
| Liver cancer | | 5 | 2.55 | 1.96 (0.72-4.86) |  | 0 | 1.49 | N/A |
| Oral and pharyngeal cancer | | 3 | 0.77 | 3.92 (1.01-12.49) |  | 0 | 0.39 | N/A |
| Colorectal cancer | | 1 | 3.77 | 0.27 (0.01-1.75) |  | 2 | 2.36 | 0.85 (0.15-3.43) |
| Esophageal cancer | | 2 | 1.40 | 1.43 (0.25-5.77) |  | 0 | 0.64 | N/A |
| Renal and urinary tract cancer | | 1 | 0.80 | 1.25 (0.07-8.12) |  | 1 | 0.47 | 2.13 (0.11-13.83) |
| Gastric cancer | | 1 | 4.01 | 0.25 (0.01-1.62) |  | 0 | 2.32 | N/A |
| Others | | 2 | N/A | N/A |  | 0 | N/A | N/A |
| CI, confidence interval; CP, chronic pancreatitis; SMR, standardized mortality ratio | | | | | | | | |
